# Switching the World’s Salt Supply—Learning from Iodization to Achieve Potassium Enrichment

**DOI:** 10.1016/j.advnut.2023.100148

**Published:** 2023-11-15

**Authors:** Katrina R. Kissock, Greg S. Garrett, Penjani Mkambula, James D. Bullen, Kathy Trieu, Laura J. Fisher, Ellie Paige, Michael Shayne Gary, Bruce Neal

**Affiliations:** 1 The George Institute for Global Health, University of New South Wales, New South Wales, Australia; 2 Access to Nutrition Initiative (ATNI), Netherlands; 3 The Global Alliance for Improved Nutrition (GAIN), Switzerland; 4 National Centre for Epidemiology and Population Health, The Australian National University, Canberra, Australian Capital Territory, Australia; 5 Business School, University of New South Wales, New South Wales, Australia; 6 School of Public Health, Imperial College London, United Kingdom

**Keywords:** salt iodization, potassium-enriched salt, salt substitute, public health, sodium reduction, salt supply, implementation scale-up

## Abstract

Sodium is an essential dietary component, but excess sodium intake can lead to high blood pressure and an increased risk of cardiovascular disease. Many national and international bodies, including the World Health Organization, have advocated for population-wide sodium reduction interventions. Most have been unsuccessful due to inadequate sodium reduction by food industry and difficulties in persuading consumers to add less salt to food. Recent research highlights potassium-enriched salt as a new, feasible, acceptable, and scalable approach to reducing the harms caused by excess sodium and inadequate potassium consumption. Modeling shows that a global switch from regular salt to potassium-enriched salt has the potential to avert millions of strokes, heart attacks, and premature deaths worldwide each year. There will be many challenges in switching the world’s salt supply to potassium-enriched salt, but the success of universal salt iodization shows that making a global change to the manufacture and use of salt is a tractable proposition. This in-depth review of universal salt iodization identified the importance of a multisectoral effort with strong global leadership, the support of multilateral organizations, engagement with the salt industry, empowered incountry teams, strong participation of national governments, understanding the salt supply chain, and a strategic advocacy and communication plan. Key challenges to the implementation of the iodization program were costs to government, industry, and consumers, industry concerns about consumer acceptability, variance in the size and capabilities of salt producers, inconsistent quality control, ineffective regulation, and trade-related regulatory issues. Many of the opportunities and challenges to universal salt iodization will likely also be applicable to switching the global salt supply to iodized and potassium-enriched salt.

**Statement of significance**

Previous research has explored universal salt iodization processes and the beneficial effects of salt iodization and potassium-enriched salt separately. This manuscript provides a novel perspective of applying the mechanisms and lessons learned from successful universal salt iodization to the scale-up of potassium-enriched salt globally.

## Background

Sodium, usually in the form of salt (sodium chloride), is an essential dietary component. However, although sodium is vital to human physiology, levels of sodium intake are far greater than those present during most of hominid evolution, with significant adverse health consequences [1, 2]. In particular, high blood pressure as a result of excess dietary sodium intake leads to millions of premature strokes, heart attacks, and deaths worldwide each year [3, 4].

For more than 3 decades, the WHO and many other national and international bodies [5] have advocated for clinical and public health interventions targeting sodium reduction. Most have been unsuccessful, as it has proven difficult to change dietary behavior among individuals and populations [6], and there has been little reformulation of processed foods by the food industry [7, 8]. There is no evidence that average global dietary sodium consumption has declined over this period [1], and there is little prospect that any nation will reach the 30% reduction in sodium intake target by 2025 [8]. The challenge has been identifying effective, scalable, sustainable, and affordable interventions able to deliver sodium reduction. Adverse health outcomes from excess sodium consumption remain highly prevalent, and the large health gains possible from population-wide sodium reduction remain untapped.

Increasing potassium intake is also shown to lower blood pressure, with growing evidence to suggest the combined effects of sodium reduction and potassium increase are greater than either in isolation [9]. Global intakes of potassium currently fall below the current WHO-recommended minimum intake of 3510 mg/d [10]. The Salt Substitute and Stroke Study (SSaSS) highlighted the potential for potassium-enriched salt to ameliorate the harms caused by excess dietary sodium consumption and inadequate dietary potassium consumption. This large-scale, randomized trial included 20,995 older people obtaining most of their sodium intake from salt used in the home and provided half with potassium-enriched salt (25% potassium chloride, 75% sodium chloride), whereas the remainder continued to use regular salt (100% sodium chloride). Over 5 y, those assigned to potassium-enriched salt had lower blood pressure and lower risks of stroke (-14%, *P* = 0.006), major cardiovascular events (-13%, *P* < 0.001), and premature death (-12%, *P* < 0.001). No harm resulted from using the potassium-enriched salt, and it was very well tolerated, with 92% still using the potassium-enriched salt at the completion of follow-up [11].

The health benefits from potassium-enriched salt are likely to be achieved across most population groups, with recent summary analyses of all potassium-enriched salt trials showing lower blood pressure when switching to potassium-enriched salt for every population subset studied [12]. Since high blood pressure is a leading contributor to cause of death in most populations, and blood pressure lowering interventions protect diverse population groups [13], the potential health gains of switching from regular salt to potassium-enriched salt are substantial. In China, for example, it is estimated that almost one million strokes, heart attacks, and premature deaths could be averted each year if there was a nationwide switch to the use of potassium-enriched salt [14].

Furthermore, potassium-enriched salt is relatively low cost, tastes like regular salt, and is easy to use [15]. Potassium-enriched salt can simply be switched to regular salt when cooking and seasoning, with little behavior change required. Potassium-enriched salt could also be used in restaurants and as an alternative to regular salt in many food processing applications. Finally, potassium-enriched salt is an accessible resource that could be used as an initial lifestyle recommendation alongside dietary changes and adjunct to drug-based approaches to lowering blood pressure, particularly in communities where healthcare infrastructure is limited, and the costs of healthcare services are high [16].

Achieving the enormous health gains offered by potassium-enriched salt will require massive scale-up of worldwide production, distribution, and usage. This is not the first public health intervention to target the world’s salt supply, which has already been modified for the purpose of delivering adequate iodine intake. The mechanisms used for the universal salt iodization program will likely be highly relevant to efforts to change the world’s salt supply a second time to iodized and potassium-enriched salt.

### Iodization of the salt supply

**FIGURE 1:**
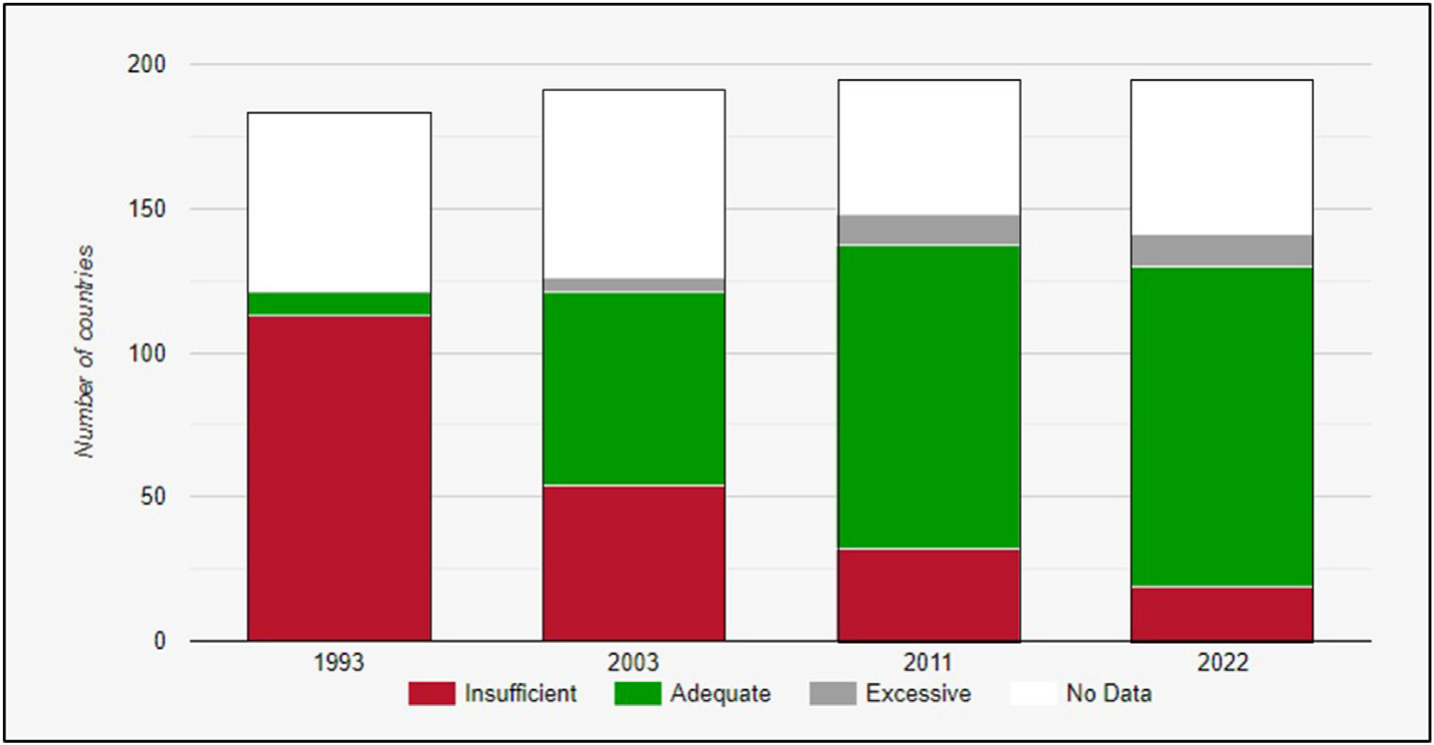
Global iodine intake status between 1993 and 2022 based on school-aged children. Figure obtained from Iodine Global Network [21] with permission.

Salt iodization is a global public health success story that has addressed the multiple serious health risks of inadequate iodine consumption for children and adults [[17], [18], [19]]. Insufficient iodine consumption during pregnancy causes low birth weight, premature birth, fetal brain damage, and increased risks of perinatal death and infant mortality. Postpartum iodine deficiency is associated with impaired cognitive and motor development, which affects a child’s performance at school as well as work productivity in adulthood. Inadequate iodine can also lead to goiter and hypothyroidism with significant associated morbidity. In 1960, it was estimated that up to 60% of the world’s population had some degree of goiter [20]. However, decades of work fortifying salt with iodine means that most countries across the world now have adequate iodine intake [21] (Figure 1).

**FIGURE 2:**
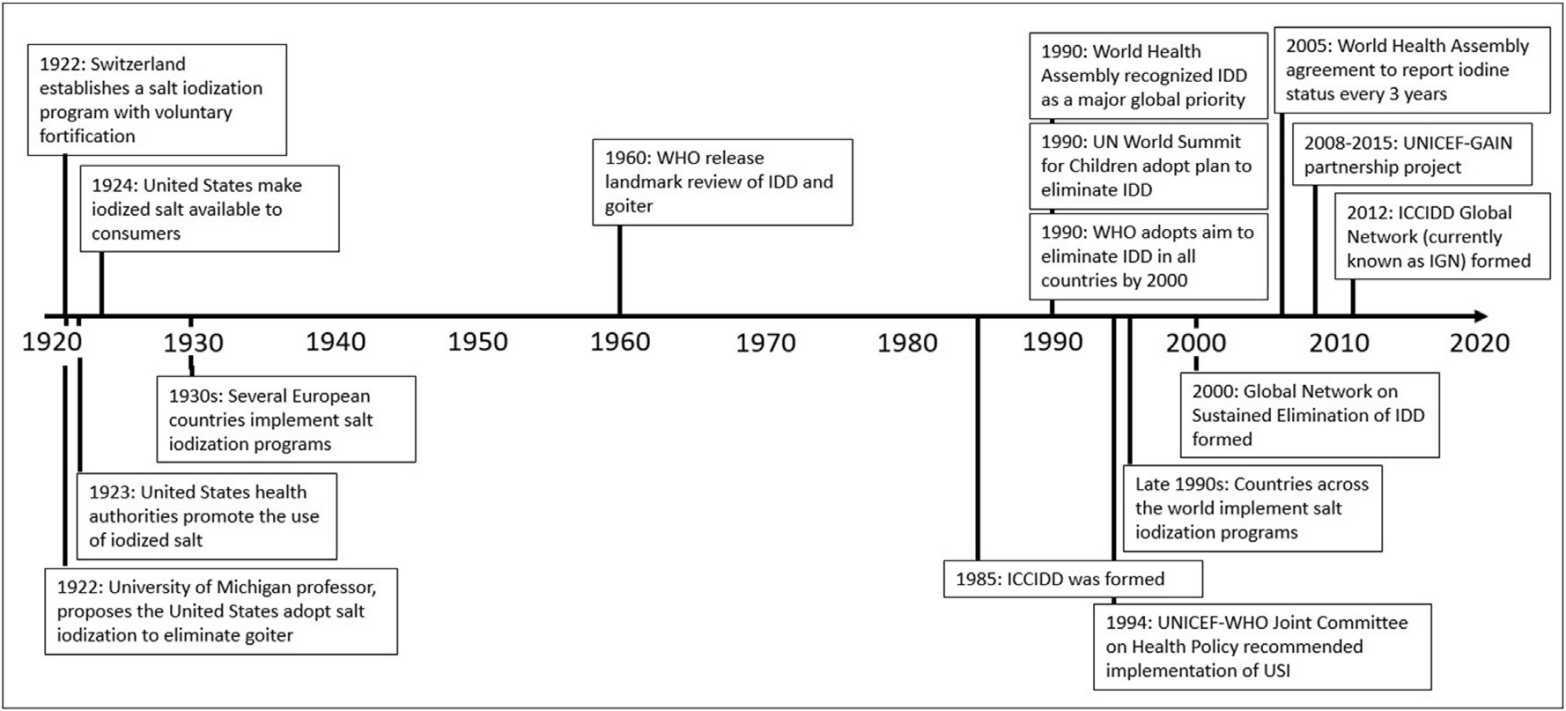
Milestones in the implementation of universal salt iodization GAIN, Global Alliance for Improved Nutrition; ICCIDD, International Council for Control of Iodine Deficiency Disorders; IDD, iodine deficiency disorder; IGN, Iodine Global Network; UN, United Nations; USI, Universal Salt Iodization.

The first country to iodize salt was Switzerland in 1922, with voluntary programs initiated over the next 2 decades in the United States and a series of European countries (Figure 2) [22, 23]. The reach and sustainability of these programs were limited by war, political unrest, and rapidly evolving trade and industry practices that frequently made implementation unsustainable [23]. In 1960, the WHO released a landmark review on goiter [20] that cataloged the scale of the problem, the severity of the health issues caused by iodine deficiency disorders (IDDs), and the clear potential for amelioration through iodine supplementation. Iodization of salt for domestic use was identified as the most convenient, practical, and likely effective method of prevention due to widespread global salt consumption, the technical simplicity of salt fortification, and the low cost of implementation. It took another 25 y for definitive action to drive forward the global iodization agenda, with the International Council for Control of Iodine Deficiency Disorders (ICCIDD) established in 1985. IDDs were then recognized as a global priority by the World Health Assembly in 1990, and plans to eliminate IDDs by the year 2000 were adopted by the WHO [24] and the United Nations World Summit for Children [25]. By the late 1990s, most countries worldwide had planned or commenced implementation of salt iodization programs [23].

There is now strong evidence of success for universal salt iodization. The number of iodine-deficient countries has fallen from 113 in 1990 to 21 in 2020 [18], and almost 90% of the global population now uses iodized salt [26]. By 2021, there were 124 countries with mandatory salt iodization requirements, and a further 21 countries had clearly defined voluntary iodization programs [18]. Multiple studies indicate large health benefits from the iodization of salt [27], though challenges with sustaining the universal salt iodization program remain.

### Key enablers of universal salt iodization

**FIGURE 3:**
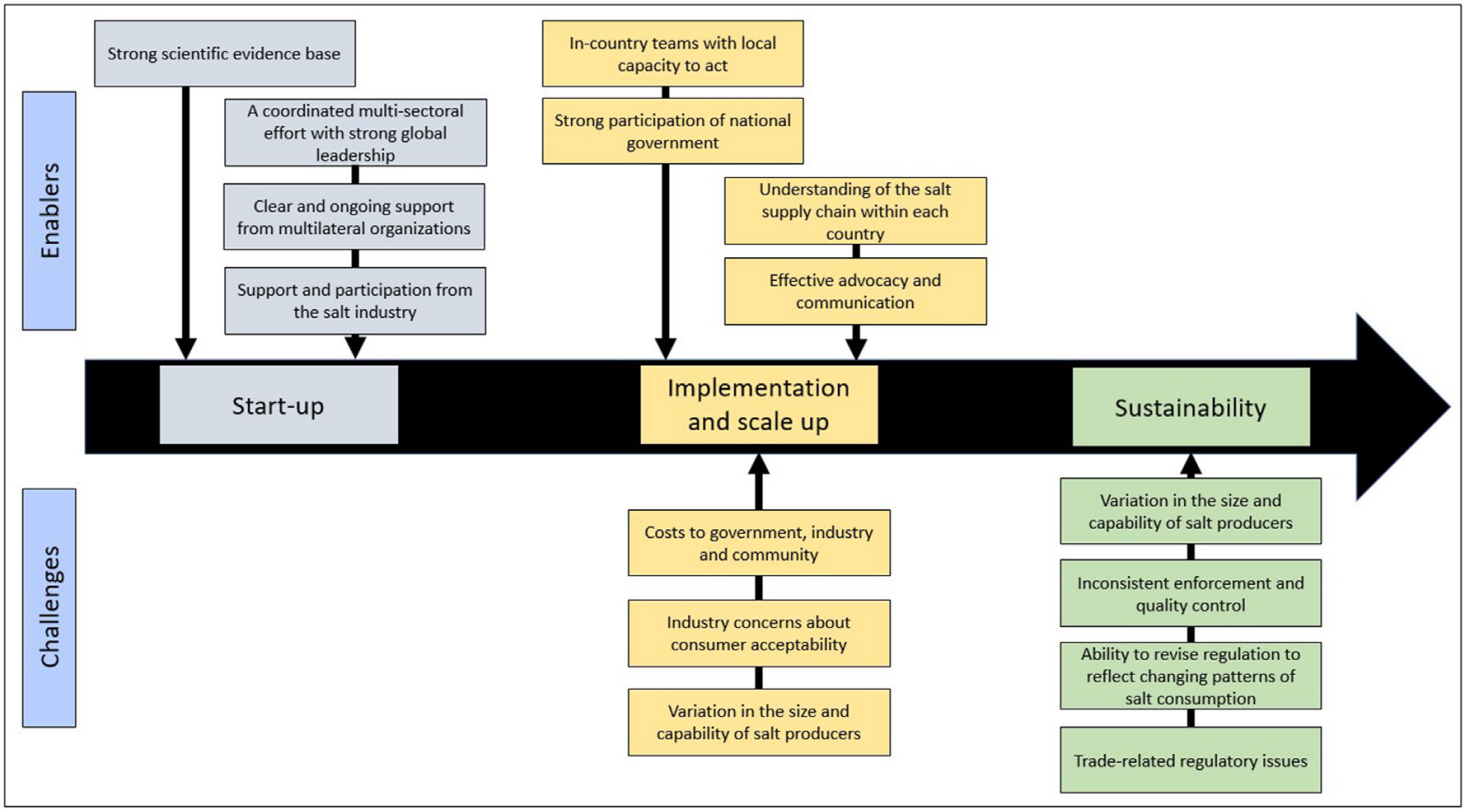
Key enablers and challenges to universal salt iodization.

The universal salt iodization program is a consequence of multiple interacting streams of work progressed over an extended period. The first 50 y of activity achieved relatively limited impact, but the last few decades have seen rapid scale-up and major health impact. Understanding the enablers and challenges should provide useful insights into switching the world’s salt supply from iodized salt to iodized and potassium-enriched salt. Insights were sought through desktop review of key literature, evaluation of organizational websites, and conversations with representatives from the Iodine Global Network and others engaged in food fortification efforts (Figure 3).

#### A coordinated multisectoral effort with strong global leadership

The ICCIDD, established in 1985, was built on the scientific evidence base for iodine deficiency and provided the leadership required for the adoption of recommendations and resolutions on iodization by the World Health Assembly, the WHO, and the United Nations. Subsequently, in 2000, the Global Network on the Sustained Elimination of Iodine Deficiency Disorders was formed. This group shifted emphasis from the primarily scientific inquiries of ICCIDD to a multisectoral implementation approach that included engagement with government, the food industry, and public health organizations. This global network formally merged with ICCIDD in 2012 to form the single ICCIDD Global Network with the goal of resolving overlaps in function and difficulties in coordinating activity between the 2 groups. Challenges with securing adequate funding to support the dual initiatives were also a key factor in driving the merger. This group was renamed the Iodine Global Network (IGN) in 2014 [28] and continues to guide the implementation and evaluation of salt iodization programs worldwide [29].

#### Clear and ongoing support from multilateral organizations

The identification of IDDs as a global priority by multilateral organizations such as the WHO, the World Health Assembly, the United Nations, and UNICEF was pivotal to achieving the global focus needed to drive universal salt iodization [[23], [24], [25], 30]. The need for objective and transparent centralized monitoring of progress was also identified early on, though it was not until 2005 that the World Health Assembly urged all member states to report on iodine nutrition status every 3 y [31]. Partnerships between multilateral organizations helped the wider universal salt iodization agenda progress by providing coordinated support networks and increased resources for implementation across the globe. A UNICEF and Global Alliance for Improved Nutrition (GAIN) partnership focused on universal salt iodization activities between 2008 and 2015, resulted in an additional 466 million individuals consuming adequately iodized salt across 13 priority countries [32]. Multilateral organizations continue to provide strong support for universal salt iodization, and the WHO has released reports on iodization in 2014 and 2022, with a recent focus on aligning universal salt iodization goals with the now well-recognized parallel need for sodium reduction [33, 34].

#### Support and participation from the salt industry

The salt industry is a key partner in universal salt iodization programs. Having the salt industry accept iodization of salt as the norm and commit to integrating iodization as part of standard operating procedures was a primary objective [35]. The US and European salt industries were the first to adopt salt iodization programs and did this prior to the formal identification of IDDs as a global health problem. Engagement of the salt industry by government and multilateral organizations, with industry representation in groups leading implementation of universal salt iodization, was also key. Motivators for engagement of the salt industry included the opportunity to be seen as positively contributing to a priority public health problem, provision of external technical and financial support, regulation, and penalties for noncompliance. In particular, mandatory iodization of salt drove market restructure and created parity across industry players [36].

Engagement with the salt industry also helped to ensure the sustained production of high-quality, adequately iodized salt [37]. Implementation was more straightforward in countries with a few large-scale salt producers compared with countries with multiple small-scale salt producers, the latter requiring novel approaches. In Cambodia, for example, > 150 small-scale salt producers joined together to establish a single system to coordinate the iodization of salt [38], and Pakistan is working toward consolidation of salt production by encouraging large-scale salt producers to buy salt from the many smaller-scale producers, to enable concentration of iodization processes to support quality control [39].

#### Incountry teams with local capacity to act

The most successful salt iodization programs have included legislation and regulation, monitoring systems, advocacy, and communication and have been multisectoral in approach, engaging diverse stakeholders throughout the process. Achieving broad national engagement requires strong local advocates who, for many countries, were highly motivated volunteer groups with deep local knowledge of community and government. These groups provided a vehicle for multilateral organizations to support local activities and build local infrastructure by providing technical and financial support. This has included provision of iodization machines and potassium iodate. Currently, the IGN is the primary support entity for new and ongoing incountry salt iodization efforts. However, whereas IGN can help to communicate the priority of iodine deficiency to national leaders and build partnerships, a sustained and strong coalition of local stakeholders is vital for incountry program success.

#### Strong participation of national government

Government leadership and ongoing government support have been central to the success of salt iodization in every country with a sustained and effective program. Government participation has taken multiple forms but usually comprises a national convening role, legislation, regulation, financing, and monitoring.

##### Convening

Recommendations from multilateral organizations were powerful tools for engaging local government [40], with visits to national government leaders by advocates from multilateral agencies key to securing participation. Local and global evidence were also important in encouraging governments to convene and act. Adoption by major nations, such as China in the Western Pacific, greatly encouraged adoption by other countries in the region.

##### Legislation and regulation

Mandating iodization, with enforcement and penalties for noncompliance, has been highly effective [41, 42]. Requirements to fortify household salt with iodine have been a frequent strategy [23], though best practice regulation encompasses all salt for human consumption, including salt within manufactured products. Nonmandated approaches are generally less effective, though countries such as the Netherlands have a successful voluntary program [23].

##### Financing

Government or other subsidies that reduced the costs to industry of establishing infrastructure for iodization were required for program implementation in many countries. Cost-recovery systems that continue to reimburse industry expenditure on potassium iodate remain important in some settings, but in many, the recurrent cost has been absorbed by industry. Avoiding additional costs for consumers during transition has also been central to the acceptability of national iodization programs.

##### Monitoring

Regular monitoring of both the implementation of industrial processes for iodization alongside national surveys of iodine intake status is the gold standard approach, which enables objective evaluation of the program and identifies process failures when they arise. In practice, monitoring programs are mostly limited in scope and frequency.

#### Understanding of the salt supply chain within each country

Knowledge of the supply chain for salt and factors affecting salt consumption in each country were crucial to effective implementation. Situational analyses were done repeatedly to provide a comprehensive understanding of factors affecting the salt industry, define monitoring strategies, quantify salt consumption, and ensure that the iodization program aligned with local circumstances [41, 43].

#### Effective advocacy and communication

Tailored advocacy and communication strategies were developed with national partners to take advantage of local circumstances. For example, in China, an annual national IDD day was introduced to disseminate messages about the importance of salt iodization to consumers, with a focus on hard-to-reach communities [40]. The Chinese Government and the local salt industry have also committed to annual “readvocacy” activities [44]. Elsewhere, nongovernmental organizations such as Kiwanis International have been advocating for salt iodization programs in multiple countries through engagement with governments, community radio stations, salt importers, consumer organizations, and public education campaigns.

### Key challenges to universal salt iodization

Key challenges to implementing and scaling up salt iodization have been costs, industry concerns about consumer acceptability, variation in the size and capability of salt producers, inconsistent enforcement, weak quality control, challenges in revising regulations to reflect changing patterns of salt consumption, and trade-related regulatory issues.

#### Costs to government, industry, and community

Costs for the establishment or ongoing implementation of iodization programs were identified as major challenges. In general, governments are unwilling to regulate if it will impose additional financial burden on industry or consumers; industry will not invest unless there is a commercial gain to be had, and consumers will reject additional expenses imposed upon them. Cost-effectiveness analyses can be helpful in supporting government decision-making, but even compelling cost-effectiveness data have been mostly insufficient for driving action [45]. Government or other third-party incentives and subsidies have been used in many countries to cover the one-off costs of salt iodization infrastructure, but evidence for the sustainability of fortification following start-up subsidy is mixed [46, 47]. It appears that sustainable iodization programs provide defined subsidies for a time-limited period while local cost-recovery mechanisms can be implemented.

#### Industry concerns about consumer acceptability

The food industry has raised concerns that the use of iodized salt within processed food products would be unacceptable to consumers due to potential instability of the iodine, discoloration of food, and deranged organoleptic properties [48, 49]. A subsequent research agenda showed that using iodized salt in processed foods did not cause adverse changes, and this issue has not been a widespread challenge [48].

#### Variations in the size and capability of salt producers

A substantial proportion of the global salt supply is derived from a relatively small number of large salt manufacturers. A smaller but still substantial proportion comes from multiple small-scale independent producers, particularly in low- and middle-income countries, that are widely geographically distributed and thereby pose a significant implementation challenge. Providing the industrial infrastructure for iodization to small-scale manufacturers is not cost-effective, and ensuring compliance with government regulation and quality control is impossible. This has been a particular challenge for Asian and African countries, where communities served by small-scale producers remain at heightened risk of iodine deficiency [40]. Neither the consolidation of national salt manufacture nor iodization of the salt produced by small-scale manufacturers at a central facility has provided a consistently feasible solution.

#### Inconsistent enforcement and quality control

Quality of salt iodization is typically defined by national legislation and is the responsibility of central government. Achieving quality implementation requires nationwide acceptance of salt iodization and adequate resources to implement monitoring. Both have proven difficult. In Vietnam, for example, prioritization of the national iodization program was downgraded, mandatory legislation was revoked, and central monitoring activities were reduced [47]. Although mandatory salt iodization was reinstated in 2016, there has been little enforcement, and Vietnam is currently designated an iodine-deficient country [50]. In general, internal monitoring of quality assurance by salt producers in conjunction with external monitoring by government is needed [41].

#### Ability to revise regulations to reflect changing patterns of salt consumption

Many regulations for the iodization of salt are focused on discretionary “table salt” used for cooking and seasoning in the home and do not cover salt used by commercial food processors or the restaurant industry [40]. With a progressive global shift toward the consumption of restaurant and processed foods there is a risk that iodine intakes will again become insufficient. Countries have been generally slow to update regulations to require iodization of salt used by the food industry. It is important that salt iodization laws are updated and enforced to ensure that all salt for human consumption is iodized, including salt used by commercial food manufacturers and the out-of-home-food sector.

#### Trade-related regulatory issues

The varied requirements for salt iodization between countries have caused trade-related concerns [40], both in terms of the requirement for iodization and the level of iodization. This can impact eligibility of products for import and export and has deterred some salt producers from engaging in iodization, where programs are voluntary. Consistency in regulation across countries was important to support international trade, availability, and intake of iodized salt.

#### Multiple fortification

Salt is a convenient vehicle for fortification [51], and there has been long-standing interest in fortifying salt with iron to combat iron deficiency [15, 52] and folic acid to reduce the prevalence of neural tube defects [53, 54]. Recently, the potential to fortify salt with potassium to decrease blood pressure and lower cardiovascular disease risk has been identified. Additional fortification has the potential to increase complexity and distract from iodization.

### The health potential of iodized and potassium-enriched salt

Iodized and potassium-enriched salt has dual potential to support global health gains by ensuring both iodine sufficiency and ameliorating the adverse effects of deranged intakes of sodium and potassium [[55], [56], [57]]. Excess sodium intake, in conjunction with inadequate potassium intake, causes high blood pressure in more than a billion adults worldwide and is responsible for millions of premature deaths each year [9, 58, 59]. Joint universal fortification of salt with iodine and potassium would see the ongoing prevention of iodine deficiency disorders for billions and additionally prevent millions of strokes and heart attacks each year.

The joint fortification of salt with iodine and potassium would also address the ‘health halo’ that iodization provides to regular salt. Regular salt fortified with iodine is often viewed as health-enhancing despite the negative health consequences of excess sodium intake [60]. Due to the harms caused by salt, the WHO has highlighted the importance of salt reduction strategies, and this has led to concern that sodium reduction strategies could lead to reduced iodine intake [61, 62]. Mechanisms for maintaining adequate iodine intake have been identified [34], but risk of reduced iodine intake from sodium reduction would be mitigated by iodizing potassium-enriched salt because the goal with potassium-enriched salt is to switch consumption, not to reduce consumption [63].

## Implications of the iodization program for a potassium-enrichment strategy

**FIGURE 4:**
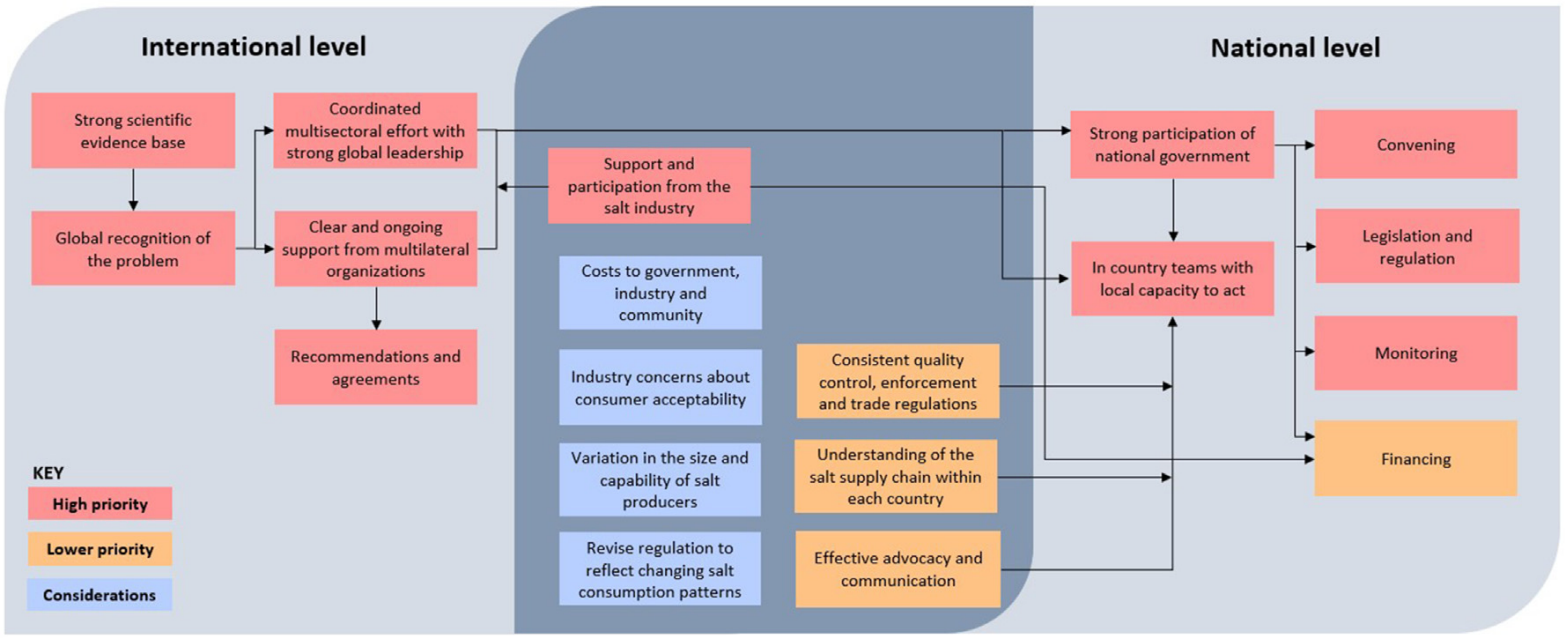
Key considerations for switching the global salt supply from iodized to iodized and potassium-enriched salt.

There are multiple features of the universal salt iodization program that seem likely to be directly applicable to a new strategy seeking to additionally fortify the global salt supply with potassium (Figure 4). Some appear likely to be of higher priority, though the importance of each will vary between countries, depending upon a breadth of local circumstances, as will the sequencing of actions required.

As for iodization, a core requirement for success will be a strong body of scientific evidence that defines the potential for potassium enrichment to deliver health gains. This is already substantively complete with complementary and coherent data from multiple sources, capped by definitive evidence of efficacy from a large-scale trial and meta-analyses [11, 12]. End-user understanding of these data is, however, inadequate, as evidenced by a Cochrane review of the trial data [64] and a recently released draft WHO guideline for the use of potassium-enriched salt (unpublished). A sound understanding of the likely balance of benefits and risks by multilateral organizations and early rectification of misconceptions will be vital for efforts to achieve potassium enrichment.  

Against a background of strong science and engagement from multilateral organizations, it will be necessary to have multisectoral incountry support for effective implementation. Strong local leadership involving government, industry, community, and other stakeholders will be key to success, with local feasibility analyses used to inform the approach most likely to be effective in each targeted jurisdiction. Initial discussions suggest that mandated requirements for potassium enrichment are unlikely to be adopted as readily as they have been for iodization in many parts of the world, reflecting shifts in citizens’ acceptance of government intervention. Although regulation may be an option in some countries, particularly where government subsidies are provided, business-focused solutions that create a new market for potassium-enriched salt seem more likely to deliver early gains in many countries. Of note, potassium-enriched salts are already available in many countries, though they constitute only a small proportion of sales in all. In addition to increasing consumer demand, there will be significant requirements for scaling of manufacture. Global production of the fortification commodity, food grade potassium chloride, will need to be hugely increased, an issue that was not faced by the iodization program. Engaging the salt industry and governments will also be vital, as will implementing effective advocacy and communication programs targeting consumers, food industry, health professionals, government, and academia.

Potassium chloride is more expensive than sodium chloride, and potassium-enriched salt is, respectively, more expensive than regular salt [65]. It is evident from the universal salt iodization program that any additional cost burden on consumers represents a significant barrier to uptake as consumers are unwilling to pay it, and governments are unwilling to impose it. There may be an opportunity for the salt industry to profit from increased sales of a higher cost commodity, but even in higher-income countries, potassium-enriched salt needs to be switched from a price-premium health product to an everyday item offered at low cost. Economies of scale introduced by large production volumes should enable costs to be brought down in most settings. In parallel, promotion of the health benefits of potassium-enriched salt may help some sectors of the community, such as those with high blood pressure, overcome the cost barrier. Potassium-enriched salt and salt reduction programs, more broadly, have shown to be highly cost-effective or cost-saving to the health care system [66], and these economic benefits may provide a medium-term opportunity for engaging governments in discussions about price subsidy.

Variations in the dietary sources of salt between countries will also influence the focus of national potassium-enrichment strategies. In countries where salt used in the home is the primary source of dietary salt intake, targeting the “table” salt provided by retail outlets will be key. In other countries where most dietary salt derives from packaged foods and foods eaten outside the home, the food processing industry, restaurants, and ingredient suppliers will be central to implementation efforts. As for iodization strategies, interventions that seek to upgrade the entire salt supply are likely to be the most effective and may also be easier to implement and maintain because the number of salt manufacturers and importers is typically much smaller than the number of food manufacturers, retailers, and restaurateurs.

The universal iodization program has benefited from its focus on females and children as the main victims of inadequate iodine consumption and the primary beneficiaries of adequate iodine intake since iodine is essential for early brain development during pregnancy and childhood. Achieving the same support for potassium enrichment of salt will require a different advocacy strategy because the cardiovascular consequences occur mostly in older adults who have not, until recently, been the focus of most multilateral agencies and high-profile funders. Nonetheless, the disease burden avoidable with potassium-enriched salt is also very large, and a strong case has already been made for salt reduction more broadly. The feasibility of scaling potassium-enriched salt compared with other salt reduction strategies is an important differentiator and could be a focus of promotion activities. The current geopolitical environment may also present challenges that were not experienced during the scale-up of salt iodization. In particular, the current largest producers of potash, the mineral from which most potassium chloride is sourced, are Russia and Belarus, where commodity prices have spiked recently. In addition, there was a strong spirit of global cooperation achieved around the universal iodization program, and this may be difficult to replicate in the present socio-political environment.

Challenges that the iodization program faced relating to consumer and industry distrust of fortified ingredients and possible adverse impacts on the quality of finished products are also likely to be a problem for potassium-enriched salt. Potassium enrichment has been reported to result in negative organoleptic properties, and in some cases, differences can be detected by expert tasters. However, taste issues are infrequently reported by general community consumers [11, 67], with widespread acceptability of salts containing up to 30% potassium chloride. Regardless, organoleptic concerns are widely held and will act as a barrier to uptake by both industry and consumers.

Consumers can also be wary of additives to foods and beverages, with fluoridation of water, for example, vigorously campaigned against despite large public health benefits and no substantive evidence of harm [68]. Potassium-fortification is likely to be challenged in the same way by a subset of consumers. Industry can also be reluctant to incorporate new components that add even minor additional complexity to the product label because of possible adverse consumer perceptions and the subsequent additional work for manufacturing processes.

Perceptions of health risk are already more challenging for potassium enrichment compared to iodization because of the increased risk of hyperkalemia in patients with impaired kidney function or those taking medicines that raise blood potassium levels. There is a strong theoretical basis for concern among individuals who are already recommended to avoid foods high in potassium. Whereas harms have not been shown to date in any trial of a potassium-enriched salt [11, 12], it may be that promotion in clinical hypertension management settings may be an early focus, where risks of misuse can be minimized, alongside community education about risk and benefits, and a systematic and standardized provision of advice on product labels.

In conclusion, analysis of the universal salt iodization program has provided valuable insight into the main challenges that will face a potassium-enrichment program and identified a series of potential amelioration strategies. Although there will clearly be significant challenges to achieving a second global upgrade to the global salt supply, the experience of the universal salt iodization program suggests that it should be a tractable proposition.

## Acknowledgments

The authors acknowledge Jan Werner Schultink for providing technical knowledge and support in the context of universal salt iodization. Jan Werner Schultink also reviewed the manuscript.

### Author contributions

The authors’ contributions were as follows—KRK, EP, and BN designed the research; KRK conducted research; and KRK, GSG, PM, JDB, KT, LJF, EP, MSG and BN wrote the manuscript. KRK had primary responsibility for content. All authors have read and approved the final manuscript.

### Conflict of interest

The authors report no conflicts of interest.

### Funding

The authors report no funding received for this study.
